# Rectal Dieulafoy Lesions: A Rare Etiology of Chronic Lower Gastrointestinal Bleeding

**DOI:** 10.1155/2014/180230

**Published:** 2014-10-01

**Authors:** Ugur Dogan, Ismail Gomceli, Umit Koc, Mani Habibi, Nurullah Bulbuller

**Affiliations:** Department of General Surgery, Antalya Education and Research Hospital, Muratpaşa, 07070 Antalya, Turkey

## Abstract

Dieulafoy lesion is rarely seen, yet it can be life-threatening. This lesion makes up to 1-2% of gastrointestinal bleedings and must definitely be considered in gastrointestinal bleedings whose source cannot be identified. In this case study, the 75-year-old woman was suffering from active, fresh, and massive rectal bleeding. Colonoscopy was applied in order to find out the source of bleeding. In the typical endoscopic appearance of the lesion a single round mucosal defect in the rectum and arterial bleeding were observed. To procure hemostasis, epinephrine was injected into the lesion and the bleeding vein was sutured.

## 1. Introduction

The incidence of acute gastrointestinal bleeding is 50–150/100000. 80% of these patients constitute the group suffering from peptic ulcer, esophageal erosion, or gastroduodenal bleeding [1]. 5% of these bleedings is occult gastrointestinal bleeding. The detection of occult bleeding is difficult and endoscopic intervention and barium studies might be necessary for diagnosis. Dieulafoy lesion is one of the causes of rarely seen occult gastrointestinal bleedings, and it can be life-threatening as its diagnosis is difficult [2, 3].

Dieulafoy lesion was first described by Gallard in 1884 as aneurysms of the stomach [3, 4]. The French Surgeon George Dieulafoy identified Dieulafoy lesion in 1898 in three patients with massive upper gastrointestinal bleeding [3].

In Dieulafoy lesion the histopathological vascular structure is normal, yet the vessel diameter is large [2, 3]. Small mucosal defect and fibrinoid necrosis are observed in the lesion [5]. In the literature, the location of Dieulafoy lesion has been identified by 71% in the stomach, 8% in the esophagus, 15% in the duodenum, 1% in the jejunum-ileum, 2% in the colon, 2% in the rectum, and 1% in the gastric anastomosis [5, 6]. Acute, massive, and recurrent gastrointestinal bleedings are present in these patients. Bleedings can be in the form of hematemesis, melena, and fresh bleeding from the rectum.

## 2. Case Presentation

**Figure 1:**
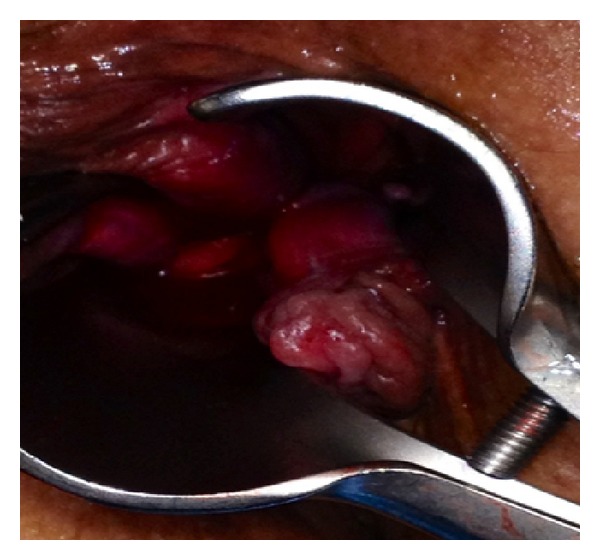
Dieulafoy lesion with fresh bleeding in anal canal.

A 75-year-old female patient who had been followed up in the nephrology clinic with the diagnosis of chronic kidney disease was referred to our clinic because of initial acute and massive rectal bleeding. A detailed assessment of the lesion could not be made by colonoscopy because of the rectum was dirty and filled with blood clot. The patient had some accompanying diseases which were hypertension, diabetes mellitus, and chronic renal insufficiency. She had no history of gastrointestinal system bleeding. Her heart rate was 120 beats per minute, systolic blood pressure was 80 mmHg, hemoglobin values with blood transfusions were 9–11 g/Ld, and hematocrit values were between 28 and 34. The abdominal region was found to be highly sensitive without any rebound during abdominal examination. Bright fresh blood was seen in the rectal examination and no mass was detected in the rectal palpation. The patient was operated on for an evaluation under anesthesia, since bleeding continued and colonoscopic diagnosis could not be made. At a distance of approximately 5-6 cm from the anal verge, an active Dieulafoy lesion was observed. A 2 mL (1/1000) epinephrine injection was applied to the lesion, and following the inability to control bleeding the active bleeding vessel was sutured. Following the hemostasis, the patient was followed up in the intensive care unit because of the comorbidities. After recovery, the patient was discharged from intensive care unit at postoperative 2th day (Figure 1).

## 3. Discussion

Dieulafoy lesion is one of the well-defined causes of acute massive gastrointestinal bleedings.

Even though it was defined in the beginning as the aneurysm of the gastric submucosal arteries, in the following years it was shown that this lesion can be seen in the whole gastrointestinal system. Typically, patients with Dieulafoy lesion are old, and this lesion is twice common in men. Patients are usually those with multiple comorbidity reasons like cardiopulmonary dysfunction and chronic renal insufficiency, and they have no serious gastrointestinal problems yet recurrent gastrointestinal bleedings. These patients also have a history of recurrent nonsteroid anti-inflammatory drugs utilization, like acetylsalicylic acid and warfarin [3, 5].

When the pathology of Dieulafoy lesions is examined, aneurysm, atherosclerosis, arthritis, and inflammation are observed in the lesions [2, 5].

One theory of spontaneous bleeding is that submucosal venous pulsation damages the epithelium and leads to local ischemia. Erosion and vascular rupture follow this pathology. Another theory is arterial thrombosis which is resulted with necrosis and bleeding. It has been shown that also the solid content in the intestine and rectum causes the development of mucosal ulceration and submucosal artery dilatation, and these result in bleeding. Furthermore, mucosal atrophy dependent on old age contributes to this process [3, 7].

Endoscopy is gold standard in the diagnosis of Dieulafoy lesion. The endoscopic diagnostic criteria of Dieulafoy lesion are as follows: (1) normal mucosa around the small defected mucosal lesion which has active pulsative bleeding smaller than 3 mm, (2) the presence of protruded vein, and (3) the observation of fresh clot attached to mucosal defect [5, 8]. Diagnosis can be made in 70% of the patients with the first endoscopy [9]. Poor visualization of the intestinal mucosa due to blood, food, or faeces in the gastrointestinal system or the presence of a hematoma may prevent the endoscopic visibility of the focal point of the bleeding. Lesions at the first 1-2 cm of the anal channel may not be seen with rectoscopy. In such cases, detailed evaluation of the focal point of the bleeding during the operation will be more effective with regard to diagnosis and treatment. When the endoscopic method is not successful, angiography can be used for diagnosis especially in colon and rectum bleedings.

The observation of curved and bleeding artery is typical in angiography [3, 5]. In the anorectal lesions, the observation of the internal iliac artery might be necessary. In the diagnosis, the bleeding focus can be identified with computerized tomography (CT) angiography [10].

The success rate in the endoscopic treatment of Dieulafoy lesions is 90% [8, 9]. Endoscopic treatment covers thermal electrocoagulation, local epinephrine injection and sclerotherapy, and mechanic band and clip application [2, 8, 11, 12]. Endoscopic ultrasound (EUS) might be helpful in detecting aberrant submucosal vessel [5].

Angiography and embolization can be practiced on patients whose treatment cannot be achieved via endoscopic methods and who are not suitable for surgery. However, it should be remembered that embolization has also the risk of ischemia.

Surgical resection might be necessary in cases resistant to endoscopic and angiographic methods [3]. Wedge resection is preferred in surgical treatment [5, 9]. Laparoscopic resection has successfully been practiced in some cases of jejunum and stomach Dieulafoy lesions [13].

The risk of recurrent bleeding of the Dieulafoy lesion has been reported as 9–40% [5, 9]. Recurring bleedings because of incomplete embolization and collateral circulation have been reported [14]. In recurrent bleedings endoscopic hemostasis could be primarily preferred.

With the developments in endoscopy, the diagnosis and treatment of Dieulafoy lesions have been successfully furthered and mortality rate has been decreased to 8,6% [14]. The comparison of mechanic methods (band and clip) to epinephrine injection has shown that the recurrence rate of bleeding is much lower [8].

Gimeno-Garcia et al. have successfully practiced the epinephrine and hemoclip application, and have designated this as the Gimeno-Garcia technique [15]. In colonic and rectal Dieulafoy lesions hemoclip is a reliable and successful method.

In our case, there was a life-threatening massive rectal bleeding in the patient. Colonoscopic evaluation could not be successful. Epinephrine injection and vein suturation were practiced under general anesthesia. The patient was followed up in the intensive care unit after active bleeding stopped. Bleeding did not recur during the follow-up period.

Considering that there might be Dieulafoy lesion in patients with massive gastrointestinal bleeding and occult bleedings, like in the case of the patient in the present study, the application of endoscopic methods must be primarily preferred in diagnosis and treatment. In cases where endoscopic method is ineffective, especially as seen in cases where the lesion is at the initial part of the anal canal, it should not be forgotten that it might be necessary to make a detailed evaluation of the focal point of bleeding during the operation.
